# Co-treatment with Esculin and erythropoietin protects against renal ischemia–reperfusion injury via P2X7 receptor inhibition and PI3K/Akt activation

**DOI:** 10.1038/s41598-022-09970-8

**Published:** 2022-04-14

**Authors:** Walaa H. El-Maadawy, Marwa Hassan, Ehab Hafiz, Mohamed H. Badawy, Samir Eldahshan, AbdulRahman AbuSeada, Maha A. M. El-Shazly, Mosad A. Ghareeb

**Affiliations:** 1 Pharmacology Department, Theodor Bilharz Research Institute, Kornaish El Nile, Warrak El-Hadar, Imbaba, P.O. Box 30, Giza, 12411 Egypt; 2 Immunology Department, Theodor Bilharz Research Institute, Warrak El-Hadar, Imbaba, P.O. Box 30, Giza, 12411 Egypt; 3 Electron Microscopy Department, Theodor Bilharz Research Institute, Warrak El-Hadar, Imbaba, P.O. Box 30, Giza, 12411 Egypt; 4 Urology Department, Theodor Bilharz Research Institute, Warrak El-Hadar, Imbaba, P.O. Box 30, Giza, 12411 Egypt; 5 Anesthesia Department, Theodor Bilharz Research Institute, Warrak El-Hadar, Imbaba, P.O. Box 30, Giza, 12411 Egypt; 6 Medicinal Chemistry Department, Theodor Bilharz Research Institute, Warrak El-Hadar, Imbaba, P.O. Box 30, Giza, 12411 Egypt

**Keywords:** Drug discovery, Molecular biology, Urology

## Abstract

Renal ischemia/reperfusion (RI/R) is a critical clinical outcome with slightly reported improvement in mortality and morbidity. Effective therapies are still crucially required. Accordingly, the therapeutic effects of esculin (ESC, LCESI-MS/MS-isolated compound from *Vachellia farnesiana* flowers extract, with reported P2X7 receptor inhibitor activity) alone and in combination with erythropoietin (EPO) were investigated against RI/R injury and the possible underlying mechanisms were delineated. ESC and EPO were administered for 7 days and 30 min prior to RI, respectively. Twenty-four hour following reperfusion, blood and kidney samples were collected. Results revealed that pretreatment with either ESC or EPO reduced serum nephrotoxicity indices, renal oxidative stress, inflammatory, and apoptosis markers. They also ameliorated the renal histopathological injury on both endothelial and tubular epithelial levels. Notably, ESC markedly inhibited P2X7 receptors and NLRP3 inflammasome signaling (downregulated NLRP3 and Caspase-1 gene expressions), whereas EPO significantly upregulated PI3K and Akt gene expressions, also p-PI3K and p-Akt levels in renal tissues. ESC, for the first time, demonstrated effective protection against RI/R-injury and its combination with EPO exerted maximal renoprotection when compared to each monotherapy, thereby representing an effective therapeutic approach via inhibiting oxidative stress, inflammation, renal tubular and endothelial injury, apoptosis, and P2X7 receptors expression, and activating PI3K/Akt pathway.

## Introduction

Renal ischemia reperfusion (RI/R) injury is an inevitable complication of perioperative acute kidney injury (AKI), including major vascular, cardiac and hepatic surgeries, and kidney transplantation. RI/R results from the disruption of renal blood flow (ischemia), followed by subsequent reperfusion^1^. This causes a sustained low local tissue oxygen supply and demand, and the accumulation of toxic byproducts from injured renal cells, leading to renal dysfunction^2^. The pathogenesis is multifactorial, including oxidative stress, inflammation, renal tubular apoptosis, and necrosis, and is mediated by a myriad of interconnected molecular pathways, and cellular mediators and regulators^3^. Among the key driving pathogenic pathways are NLRP3 inflammasome, via the uncontrolled activation of purinergic receptors (P2Rs) mainly P2X7Rs^4^ and nuclear factor (NF)-κB^5^, as well as phosphatidylinositol 3-kinase (PI3K)/serine-threonine kinase (Akt)^6^.

In view of this multifactorial nature of RI/R injury, there is a crucial necessity for the identification of effective therapeutic approaches to minimize renal tissue damage^7^. Esculin (ESC), a coumarin derivative, has several biological activities including vasoprotective^8^, anti-inflammatory, antioxidant^9^, and anti-apoptotic effects^10^. ESC is recently reported to exhibit nephroprotective activity against diabetic nephropathy in rats and LPS-induced AKI in mice via P2X7Rs inhibitory activities^11,12^. However, its protective activities against RI/R have not been previously examined. In this study, ESC was isolated from *Vachellia farnesiana* flowers, family *Fabaceae*^13^, a widely spread shrub in tropical and sub-tropical zones worldwide^14^. Traditionally, *Vachellia farnesiana* has been used for the treatment of numerous ailments as malaria and tuberculosis^14,15^.

On the other hand, erythropoietin (EPO), a hematopoietic hormone produced mainly by the kidney^16^, is reported to protect against RI/R injury^17–19^ through suppressing tubular cell apoptosis, inflammation and oxidative stress^20^. The physiological effects of erythropoietin are reported to be mediated through binding to erythropoietin receptors (EPORs)^21^. Under ischemic conditions, the binding of EPO to EPORs triggers the activation of multiple signaling cascades including PI3K/Akt^22^ and signal transducer and activator of transcription-3^23^ signaling pathways. Nevertheless, large dosage are required to achieve renoprotection, which is often associated with adverse events including renal fibrosis, hypertension, and thrombosis, thereby limiting its clinical applicability^24–27^.

Not only, have controversial outcomes from clinical trials still debated the limited effects of EPO alone in kidney transplant patients^28,29^, but also its safety when administrated in high dose^30^. Several preclinical studies indicated that EPO when co-administered with other drugs such as vitamin D, melatonin or *N*-acetyl cysteine, achieved maximal renoprotection against RI/R injury in comparison to EPO alone^20,31,32^.

Accordingly, in this study we aimed to evaluate the possible nephroprotective effects of ESC alone, and its synergistic effects when combined with EPO against RI/R injury in rats, while focusing mainly on their effects on P2X7Rs, as well as NLRP3 inflammasome and PI3K/Akt pathways.

## Results

### Chemical characterization and chromatographic isolation

**Figure 1:**
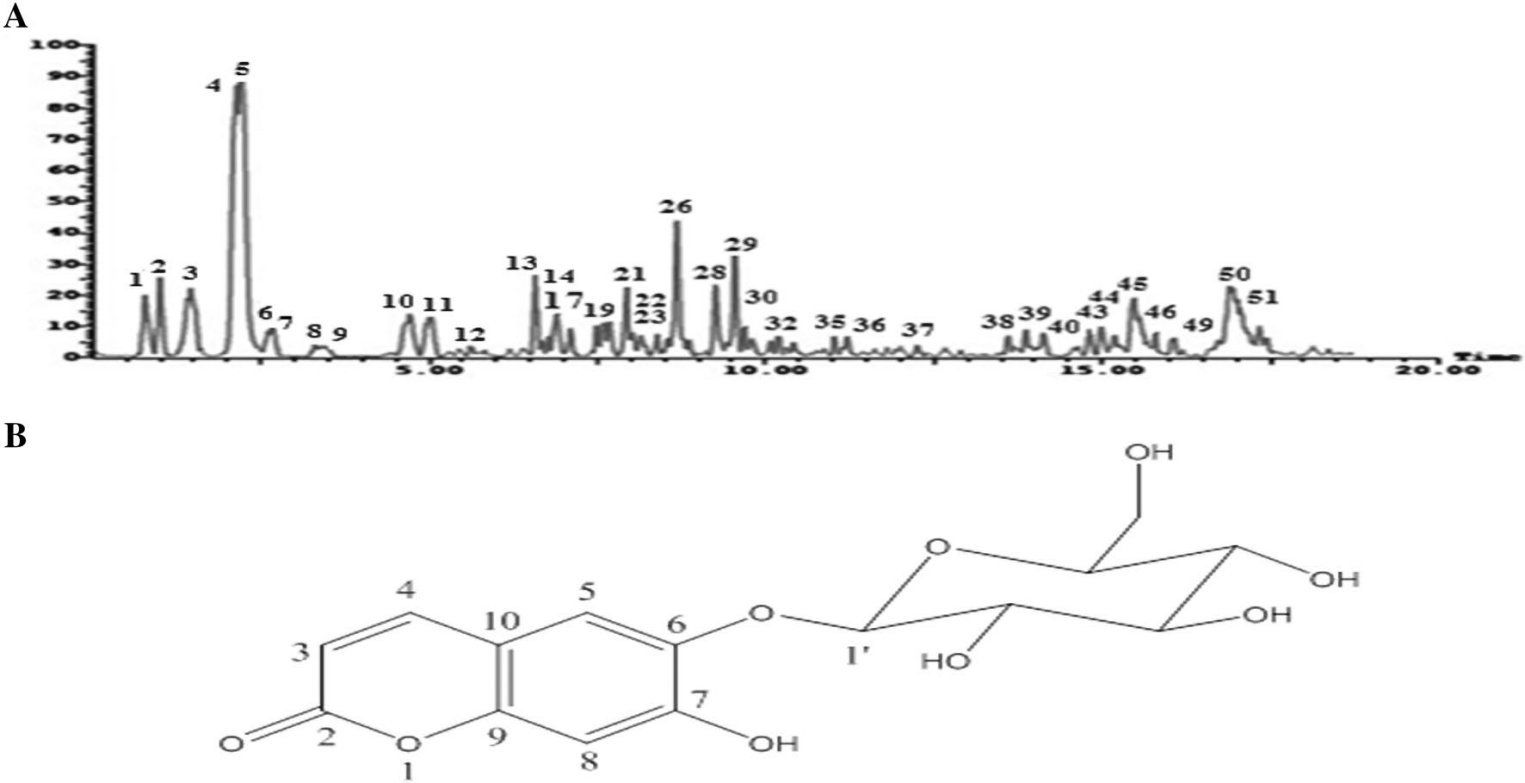
(**A**) Negative LC–ESI–MS/MS profile of phenolic compounds from ethyl acetate extract of *V. farnesiana* flowers (Tentative identification of the compounds is provided in Table 2S). (**B**) Chemical structure of ESC.

Ethyl acetate extract was selected among all tested extracts for further chemical profiling and identification of its chemical constituents via using LC–ESI–MS/MS analysis as well as chromatographic isolation. This is related to its strong antioxidant activities and the high phenolic content, as shown in Table 1S. LC–ESI–MS/MS investigation of the ethyl acetate extract in negative ion mode led to tentative identification of 48 secondary metabolites were categorized as phenolic acids, phenolic acid derivatives, organic acid, flavonoides (aglycones & glycosides), coumarins, iridoids and others (Fig. 1A; Table 2S). The identification based on their retention times, fragmentation patterns, and via comparison the available reported data.

Among the identified compounds by LC–ESI–MS/MS, eight phenolic compounds were isolated. Based on physicochemical properties, CO-chromatography (CO-TLC & CO-PC), acid hydrolysis, spectral analysis (^1^H & ^13^C-NMR) and via comparison with available reported data the isolated compounds were identified as; gallic acid, methyl gallate, p-coumaric acid, quercetin, taxifolin, naringenin, quercetin 3-*O*-glucoside (Supplementary file) and ESC.

### Structural elucidation of ESC

Esculin was isolated as white amorphous crystals, m.p. 203–205 °C. Rf: (0.59; 15% AcOH) and (0.81; BAW: *n*-BuOH: AcOH: H_2_O (4:1:5; Top phase). It showed blue spot under UV-light. Acid hydrolysis gave glucose as sugar moiety and esculetin as aglycone via comparative TLC (CO-TLC) indicating its glycosidic nature as a monoglucoside. ^1^H-NMR (400 MHz, DMSO-d_6_) spectral data showed the presence of characteristic signals for aglycone moiety at δ_H_: 7.87 (d, *J* = 9.5 Hz, 1H, H-4), 7.41 (s, 1H, H-5), 6.84 (s, 1H, H-8), and 6.23 (d, *J* = 9.48 Hz, 1H, H-3). Also, the anomeric proton of glucose moiety was observed at δ_H_ 4.79 (d, *J* = 7.2 Hz, 1H, H-1′) confirmed its glycosidic nature. ^13^C-NMR (100 MHz, DMSO-d_6_) showed the presence of fifteen catbon-13 signals including aglycone carbon signals at δ_C_: 161.0 (C-2), 112.54 (C-3), 144.94 (C-4), 115.07 (C-5), 151.79 (C-6), 143.12 (C-7), 103.60 (C-8), 150.89 (C-9), and 111.20 (C-10). While sugar carbons were resonated at δ_C_: 103.60 (C-1'), 74.72 (C-2′), 77.71 (C-3′), 70.16 (C-4′), 76.48 (C-5′), and 61.11 (C-6′). Based on physicochemical examination, matched LC-ESI-MS/MS peak, acid hydrolysis and spectral analysis (^1^H & ^13^C-NMR) it was identified as esculetin 6-*O*-glucoside (ESC) (Fig. 1B).

### Effects of pretreatment with ESC, EPO, and their combination on RI/R renal injury

**Figure 2:**
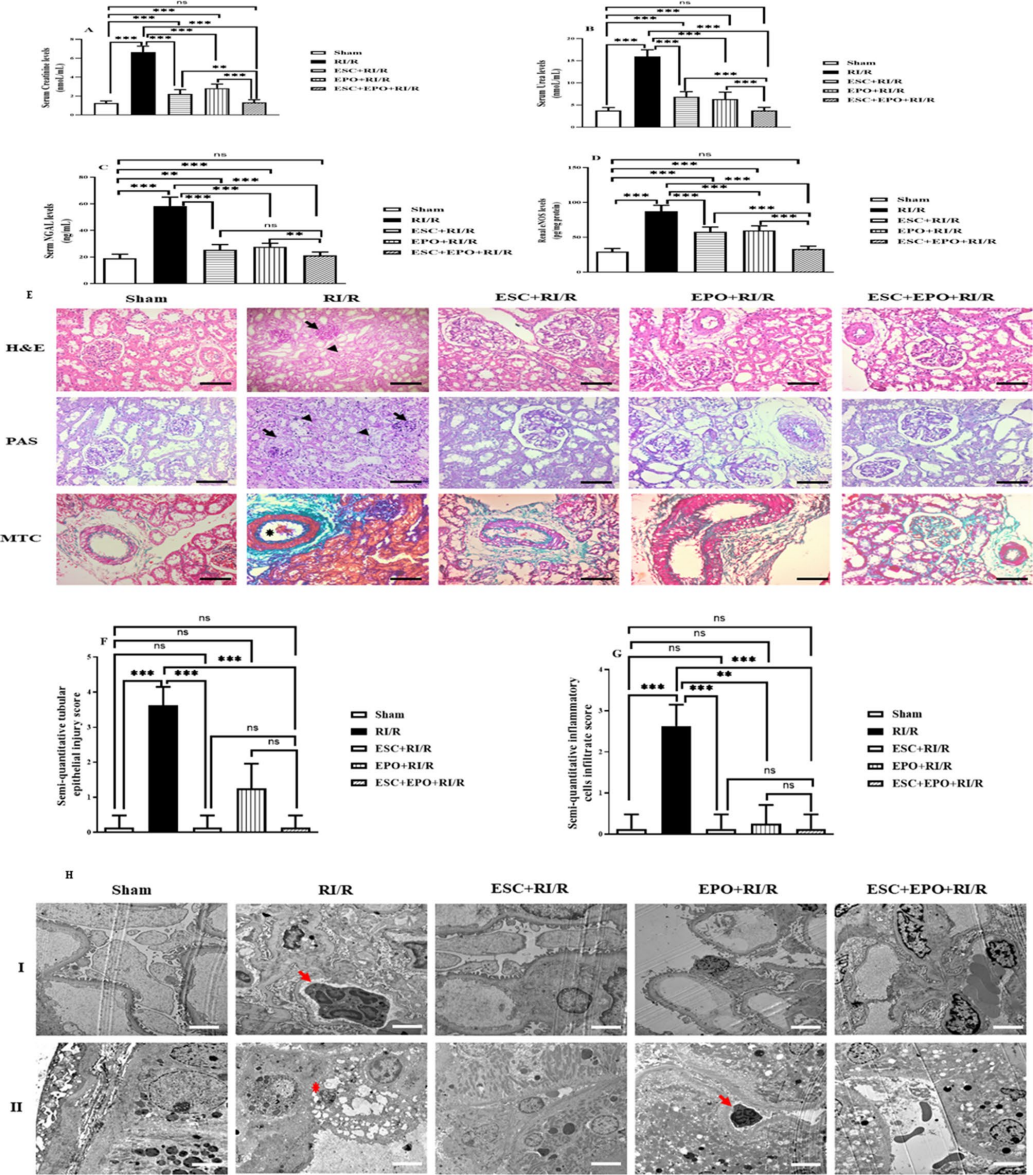
Co-treatment with ESC and EPO counteracted RI/R-induced injury. (**A**) Creatinine, (**B**) urea and (**C**) KIM-1 Serum levels, and (**D**) eNOS renal levels. (**E**) Representative photomicrographs showing histological assessment of RI/R injury stained with H&E, PAS and MTC (× 200, scale bar 50 µm, black arrow: glomerular tuft collapse, arrow heads: tubular injury with epithelial vaculation and coagulative necrosis, asterisk: red blood cells and fibrin emboli). Semi-quantitative estimation of histopathological score for (**F**) renal tubular epithelial injury and (**G**) inflammatory cells infiltration. (**H**) Photomicrographs of ultrastructural assessments of RI/R injury using TEM (I: Glomerular capillary tuft, II: Tubules and peritubular capillaries, × 5000, scale bar 2000 nm), red arrows: represent neutrophil infiltrate within glomerular tufts in RI/R group and was only noted in the peritubular capillaries of the EPO pretreated group, red asterisk: represents tubular epithelial apoptotic changes in RI/R group illustrated by nuclear pyknosis, cytoplasmic vacuolation and sequestration. Results are expressed as mean ± SD (n = 8), *p < 0.05, **p < 0.01, ***p < 0.001, ns: non-significant.

Rats subjected to RI/R injury demonstrated a pronounced elevation in serum levels of nephrotoxicity markers (creatinine, urea, and NGAL) and a substantial reduction in renal levels of the endothelial injury marker, eNOS, when compared with sham group. Pretreatment with either ESC or EPO resulted in a marked reduction in the serum levels of nephrotoxicity markers and a considerable increase in eNOS renal levels as compared with RI/R group. However, combination of ESC and EPO restored the levels of serum creatinine, urea, and NGAL, and renal eNOS (Fig. 2A–D).

These results were complemented with the histopathological analysis (Fig. 2E–G), where hypoxemic injury to the renal parenchyma was clearly evident in the renal tissues of rats subjected to RI/R injury as compared with sham group. This was manifested in the form of marked tubular injury involving proximal tubules with whole spectrum from cytoplasmic vacuolation up to focal necrosis. The majority of glomeruli were shrunken with occasional segmental tuft collapse, focal congestion, and frank necrosis was also noted. In addition, endothelial injury with formation of red cell emboli and early fibrin deposition were detected. The interstitium showed mild to moderate edema with hemorrhagic foci and inflammatory infiltrate mainly neutrophils. Meanwhile, pretreatment with EPO showed protective effects on the microvascular network including glomeruli and peritubular capillaries guarding against the development of thrombotic microangiopathy, as well as markedly decreased inflammation. Additionally, it exhibited significant protective effects on the vascular smooth muscle cells with patent arteries and arterioles, and preserved endothelium. However, its protective effect on tubular epithelium was limited, represented by focal moderate injury, as well as, focal apoptosis with desquamation of cells into the lumen. Noticeably, ESC pretreatment resulted in prominent protective effects on both tubular epithelium and vascular endothelial levels. It also showed guarding effect against inflammation. The combined therapy group had a comparable protective effect of EPO regarding the vasculature combined with tubular protective effect of ESC.

In TEM analysis (Fig. 2H), RI/R-injury was manifested within both renal tubules and glomeruli. The glomerular tufts showed diffused collapse and focal necrosis, in addition to focal podocytes’ detachment and broadening of their foot processes, and intracapillary neutrophil infiltration. The tubular epithelium showed marked injury, loss of surface microvilli, vacuolated cytoplasm, nuclear fragmentation, and apoptotic bodies’ formation. The ESC pretreated group showed patent glomerular tufts with intact capillary basement membranes mesangium and endothelium, in addition to preservation of the tubular epithelium with healthy integrated ultrastructure. Moreover, EPO pretreatment resulted in preservation of capillary loops with intact podocytes and endothelium, as well as a protective effect on the tubular epithelium, yet mild injury was still noted as cytoplasmic vacuolation and focal peritubular capillaritis. Notably, combined therapy effectively protected against tubular injury with intact healthy capillary tufts.

### Effects of pretreatment with ESC, EPO, and their combination on oxidative stress markers

**Figure 3:**
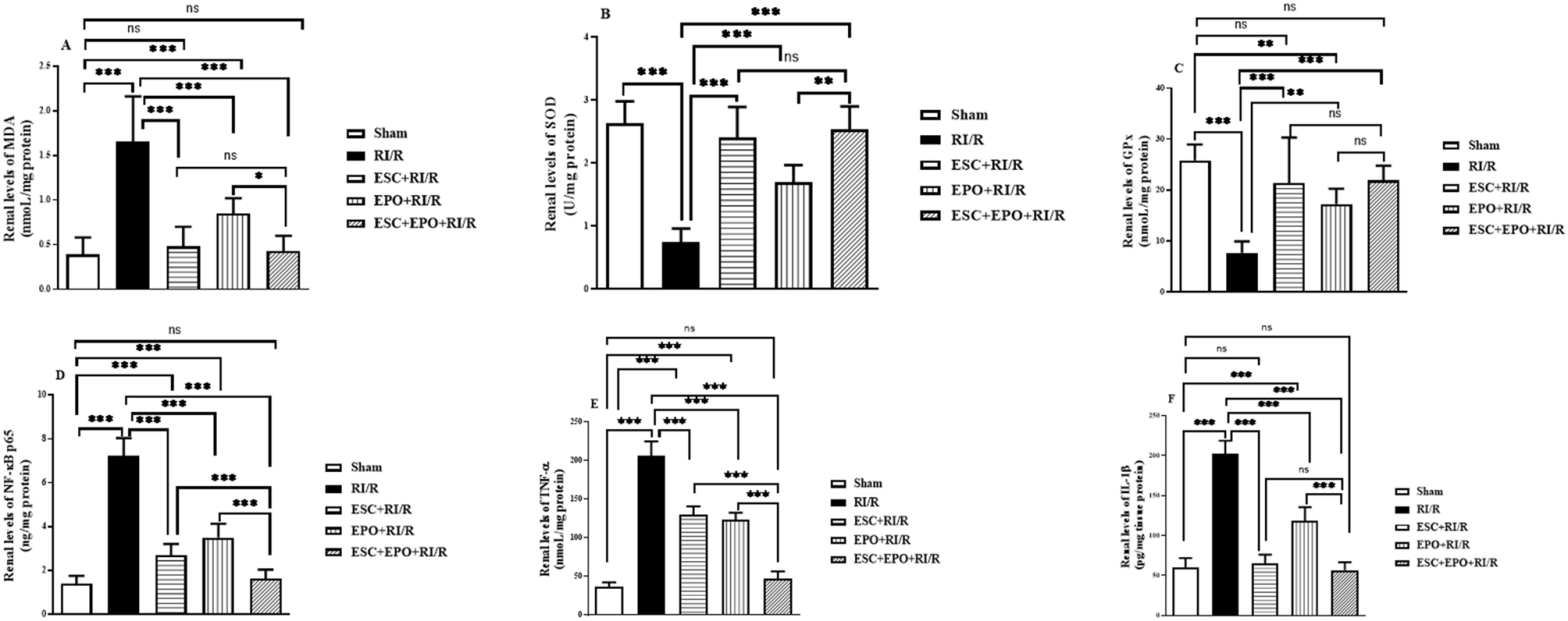
Co-treatment with ESC and EPO ameliorated oxidative stress and inflammatory markers in RI/R-induced injury. Renal levels of (**A**) MDA, (**B**) SOD, (**C**) GPx, (**D**) NF-κB p65, (**E**) TNF-α, and (**F**) IL-1β renal levels. Results are expressed as mean ± SD (n = 8), *p < 0.05, **p < 0.01, ***p < 0.001, ns: non-significant.

As shown in Fig. 3A–C, RI/R resulted in a marked increase in the renal tissue levels of MDA, accompanied by a pronounced decline in the antioxidant enzyme activity levels of SOD and GPx as compared with the sham group. EPO pretreatment significantly reduced the elevated MDA levels and raised the declined SOD and GPx renal activities when compared with RI/R group. Meanwhile, pretreatment with either ESC or the combination of ESC and EPO restored the MDA levels, and SOD and GPx activities in the injured renal tissues.

### Effects of pretreatment with ESC, EPO, and their combination on inflammatory markers

RI/R injury resulted in a pronounced elevation in the renal levels of NF-κB p65, TNF-α and IL-1β when compared with sham group. Pretreatment with either ESC or EPO significantly lowered their renal levels as compared with RI/R group, with the exception of IL-1β where ESC restored its normal levels. Notably, the renal levels of NF-κB p65, TNF-α and IL-1β reached their normal levels in the co-treated group (Fig. 3D–F).

### Effects of pretreatment with ESC, EPO, and their combination on P2X7Rs gene expression

**Figure 4:**
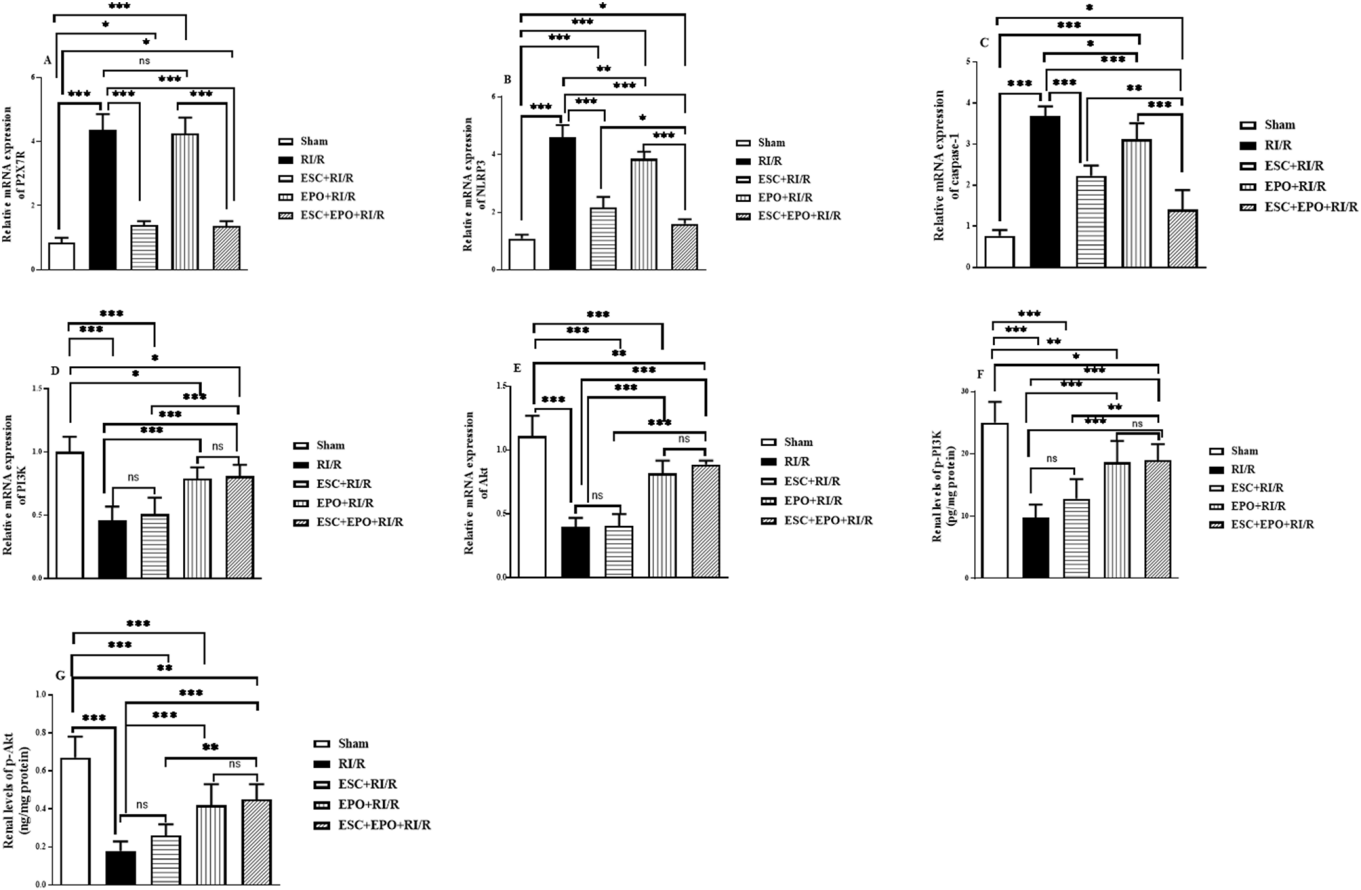
Co-treatment with ESC and EPO mitigated P2X7R, NLRP3 inflammasome and PI3K/Akt signaling in RI/R-induced injury. mRNA expression levels of (**A**) P2X7Rs, (**B**) NLRP3, (**C**) Caspase-1, (**D**) PI3K, and (**E**) Akt, and renal levels of (**F**) p-PI3K and (**F**) p-Akt. Results are expressed as mean ± SD (n = 6), *p < 0.05, **p < 0.01, ***p < 0.001, ns: non-significant.

RI/R exacerbated the mRNA expression of P2X7Rs when compared with sham group. EPO pretreatment showed no significant changes in its gene expression as compared with RI/R group. Of note, pretreatment with either EPO or combined therapy restored the normal gene expression levels of P2X7Rs (*p* < 0.01). These data indicate that the downregulatory effects on P2X7Rs were only related to ESC-pretreatment, as represented in Fig. 4A.

### Effects of pretreatment with ESC, EPO, and their combination on NLRP3 inflammasome pathway

RI/R injury induced the activation of NLRP3 inflammasome pathway as indicated by the upregulation of NLRP3 and capsase-1 mRNA expressions when compared with sham group. Pretreatment with ESC resulted in a substantial downregulation in the mRNA expression of NLRP3 inflammasome components, whereas trivial suppression in their gene expressions was observed in the EPO pretreated group when compared with RI/R group. Noticeably, Co-treatment with ESC and EPO restored the gene expression levels of NLRP3 and capsase-1 (*p* < 0.01) (Fig. 4B,C).

### Effects of pretreatment with ESC, EPO, and their combination on PI3K/Akt pathway

RI/R injury resulted in the dysregulation of PI3K/Akt signaling, as expressed by significant suppression in their gene expression levels; which was complemented with a marked decline in their phosphorylated renal levels when compared with sham group. ESC pretreatment demonstrated no significant changes in PI3K and Akt gene expression levels, whereas minor enhancement in their phosphorylated renal levels was recorded when compared with RI/R-subjected group. Conversely, pretreatment with EPO significantly upregulated PI3K and Akt gene expression as well as their phosphorylated levels when compared with RI/R injury. Meanwhile, pretreatment with combined therapy restored the normal signaling levels of PI3K/Akt (*p* < 0.01 and *p* < 0.001, respectively) in RI/R-subjected renal tissues (Fig. 4D–G).

### Effects of pretreatment with ESC, EPO, and their combination on intrinsic apoptotic pathway

**Figure 5:**
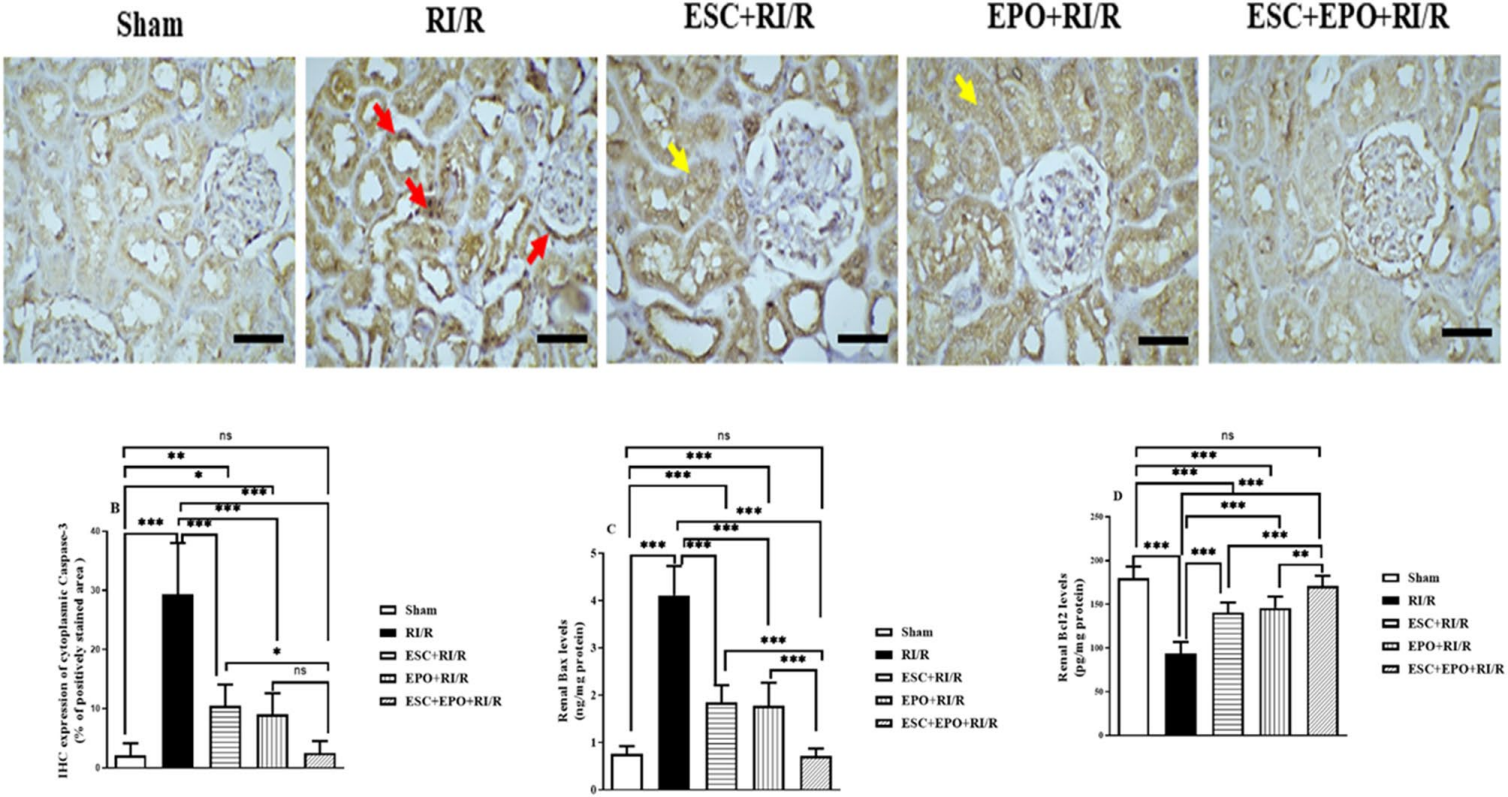
Co-treatment with ESC and EPO regulated mitochondrial apoptotic pathway in RI/R-induced injury. (**A**) Photomicrographs representing IHC-expression levels of caspase-3 (x = 400, scale bar 50 µm, red arrows: cytoplasmic and nuclear caspase-3 staining, yellow arrows: faint cytoplasmic staining cytoplasmic staining in ESC and EPO pretreated groups), (**B**) Quantitative estimation of cytoplasmic caspase-3 IHC-expression, and renal levels of (**C**) Bax and (**D**) Bcl2. Results are expressed as mean ± SD (n = 8), *p < 0.05, **p < 0.01, ***p < 0.001, ns: non-significant.

Renal sections of rats subjected to RI/R showed diffused cytoplasmic and focal nuclear caspase-3 immuno-staining within tubular epithelial cells, and focal nuclear staining in the parietal epithelial cells of Bowman’s capsule, when compared with sham group. Pretreatment with either ESC or EPO showed comparable minimal tubular cytoplasmic caspase-3 immuno-staining intensity. Impressively, the combined therapy group showed prominent protective anti-apoptotic effect with negative caspase-3 IHC-expression (Fig. 5A,B).

Furthermore, we examined the treatments effects on the renal levels of the mitochondrial apoptotic machinery-related proteins, Bax and Bcl-2. A prominent elevation in the levels of pro-apoptotic Bax, and a decrease in anti-apoptotic Bcl-2 levels were recorded in RI/R group when compared with sham group. Pretreatment with either ESC or EPO showed comparable mitigation in the bax levels and induction in the bcl-2 levels. However, the co-treatment with ESC and EPO restored their levels (Fig. 5C,D).

## Discussion

The pathophysiology of the RI/R injury is complicated and multifactorial, and mediated by multiple and interconnected molecular pathways and signaling cascades. Despite the continuous efforts for developing novel therapies that could prevent and/or treat RI/R, the mortality and morbidity of this condition has only been slightly improved in the last 4 decades, so effective therapies are crucially required^3^. Hence, in the present study, we investigated the synergistic therapeutic impact of combining ESC and EPO against RI/R injury.

In the current research work, renal ischemia was induced via bilateral clamping of renal pedicles for 45 min, followed by 24 h of reperfusion that resulted in triggering pronounced renal injury at both the functional and structural levels. This was biochemically manifested by a significant increment in serum nephrotoxicity indices including creatinine, urea, and NGAL (an early biomarker of renal tubular injury^33^. Also, endothelial dysfunction was recorded as indicated by reduced eNOS activity, which is a key enzyme in the regulation of endothelial-derived nitric oxide (NO) production, and a critical mediator in the regulation of renal blood circulation during RI/R^34^. At the histopathological level, RI/R resulted in a marked tubular injury, collapsed glomeruli, focal necrosis, and endothelial injury demarcated by the formation of red cell emboli and early fibrin deposition, meanwhile the ultrastructural examinations showed that RI/R-injury caused diffuse collapse, focal necrosis and intracapillary neutrophil infiltration within the glomerular tufts, while the tubular epithelium showed marked injury, nuclear fragmentation, and apoptotic bodies.

However, pretreatment with either ESC or EPO showed significant decline in the nephrotoxicity indices and histological improvement in RI/R-induced injury to renal tissues with more prominent effect observed in the ESC pretreated group. This could be attributed to the ability of ESC to preserve the renal tubular epithelial structure, as reported herein, where the inhibition of P2X7Rs were formerly reported to counteract the renal tubular damage during RI/R injury^5,35^. Moreover, both pretreatments exhibited comparable protective effects on the vascular smooth muscle cells with intact endothelium; they also induced comparable elevated levels of eNOS. This could be explained in the view of our reported direct suppressive effects of ESC on the gene expression of P2X7Rs, which are expressed in the endothelium and the smooth muscle layer of most of the systemic arterial and venous circulation. In concordance with our data, continuous activation of P2X7 receptors is reported to induce microvascular and endothelial dysfunction, which further promotes renal inflammation and leads to a decline in renal functions^36^, while their antagonism causes a partially NO-dependent vasodilation of the afferent, efferent, and renal arteries, thereby increasing renal perfusion^37,38^. Additionally, our reported EPO protective effects on the vascular endothelium are in coherence with previous studies, where EPO enhanced eNOS expression, improved renal function and renal hemodynamics in RI/R injury^39^. Such reported effects could emphasize the enhanced renoprotective effects of combined therapy over each monotherapy.

Oxidative stress is one of the pivotal contributors to the pathogenesis of RI/R, where re-oxygenation following the reperfusion phase leads to overproduction of reactive oxygen species (ROS) and malfunction of the antioxidant defense system (as catalase, SOD, and GPx) in renal tissues. This in turn induces lipid peroxidation, thus causing loss of renal cells' integrity, especially the renal tubular epithelial cells, endothelial dysfunction, and cell death^40^. Similarly, we recorded a marked increase in MDA levels associated with a marked drop in SOD and GPx activities in renal tissues of rats subjected to RI/R injury. Meanwhile, pretreatment with either ESC or EPO inhibited RI/R-induced oxidative stress via their antioxidant properties. Concordant with our results, the antioxidant activities of ESC were reported to improve the progression of several pathologies including renal diseases, due to its ability to scavenge ROS and inhibit neutrophil-dependent superoxide anion generation and lipid peroxidation^11^. Additionally, Liu et al.^41^ demonstrated that EPO pretreatment improved renal function and protected renal tissues from further damage in RI/R via its direct antioxidant activities as indicated by inducing the activity of antioxidant enzymes and inhibiting lipid peroxidation.

Furthermore, acute local inflammation is a hallmark of ischemic kidney injury following blood supply interruption^42^. Renal tubular epithelial cells induce overproduction of pro-inflammatory cytokines and immune responses^43^. NF-κB is the key mediator in disease progression, where its transcription participate in promoting the production of proinflammatory cytokines including TNF-α, IL-6 and IL-1β^44,45^, and its induction is mainly dependent on p65 activation^46^. Similarly, our study showed that RI/R induced the transcription of NF-κB p65 resulting in the pronounced elevation in the renal levels of TNF-α and IL-1β. Notably, co-treatment with ESC and EPO inhibited the inflammatory cascade reactions; this could be related to the early inhibition of P2X7Rs by ESC, where their inhibition have been reported to be effective against renal tubular damage and pro-inflammatory reactions during RI/R injury, as reported by Yan et al.^35^. Also, the anti-inflammatory role of EPO during RI/R is owed to suppressing pro-inflammatory cytokines production, TNF-α, IL-6, and NF-κB activation, as previously documented^47^.

Recent studies reported that under ischemic injury, P2X7R receptor signaling in renal tubular epithelial cells plays a crucial role in renal inflammation through eliciting NLRP3 inflammasome activation and subsequent inflammatory responses, thereby leading to the progression of AKI^5,48^. The activation of the NLRP3 inflammasome is mediated by two main stages^49,50^. In the initial stage, which is also known as the priming stage, the activation of NF-κB results in the upregulation of NLRP3 and pro-IL-1β expression. Then, in the second stage the release of ATP by the renal tubular epithelial cells leads to the activation P2X7Rs that triggers NLRP3 inflammasome assembly to catalyze the maturation of IL-1β. Additionally, the blockade of P2X7Rs has been demonstrated to ameliorate ischemic AKI via the inhibition of NLRP3 inflammasome activation, which resulted in the improvement of renal functions and the reduction in inflammatory cytokines production^5,48^. Likewise, in this study, markers of NLRP3 inflammasome activation were significantly upregulated, thereby triggering the renal damage induced during RI/R. Interestingly; combined therapy attenuated such renal damage via inhibting its activation, as demonstrated by restoring the normal gene expression levels of NLRP3 and caspase-1 that in turn caused inhibition in IL-1β production. Such reported effects are mainly related to ESC rather than EPO pretreatment; this could be owed to the inhibitory effects of ESC on P2X7Rs gene expression, which in turn deactivated NLRP3 inflammasome, and the production of inflammatory cytokines including IL-1β and TNF-α in rats-subjected to RI/R. These results are in coherence with those obtained by Qian et al.^5^. However, EPO showed minor inhibition in NLRP3 inflammasome activation, which is in contrary to those reported by Kwak et al.^17^.

Apoptosis of kidney tubular epithelial cells is recognized as one of the main mechanisms in RI/R, where the intrinsic apoptotic apoptosis pathway is the most important among apoptosis initiating pathways. Cellular fate depends on the imbalance between pro-apoptotic “Bax” proteins and anti-apoptotic “Bcl-2” proteins after renal RI/R, which ultimately leads to caspase-3 activation, the effector enzyme in the execution of apoptosis^3^. In our study, RI/R resulted in a significant elevation in Bax and reduction in Bcl-2 levels that led to the enhancement in caspase-3 IHC expression. However, combined therapy significantly ameliorated the mitochondrial apoptotic damage, as indicated by inhibition in IHC expression of caspase-3 and restoration of Bax and Bcl2 levels, thereby resulting in the inhibition of renal tubular cell death.

Activation of PI3K/Akt signaling is reported to negatively regulate genes involved in inflammation, thrombosis, and vascular permeability, thus protecting vascular function through activation of eNOS, and causing further increase in NO production^34^. Additionally, its activation protects kidneys from RI/R-induced injury through the inhibition of apoptosis of renal tubular cells^51^. This is achieved via Akt phosphorylation, which results in the suppression of Bax translocation to the mitochondria^52^, and restoration of the anti-apoptotic function of Bcl-2, thereby inhibiting the activation of caspase-3^53^. Herein, EPO pretreatment attenuated RI/R injury via activating PI3K, which triggered the phosphorylation of its downstream Akt to suppress the inflammatory cascade reactions, as previously reported by Zhang et al.^22^, this was associated with enhanced anti-apoptotic effects on renal tubular epithelial cells. Moreover, the improvement effects of EPO pretreatment in renal microcirculation could be also indirectly attributed to the activation of PI3K/Akt/eNOS signaling pathway. Conversely, ESC showed no effect on the activation of PI3K/Akt signaling in RI/R, thereby indicating that the effect of combined therapy on PI3K/Akt signaling is related only to EPO pretreatment.

In conclusion, our study is the first to elaborate the renoprotective effects of ESC against RI/R, which is mainly dependent on its P2X7Rs inhibitory activities. We have also delineated that co-therapy with ESC and EPO exerted prominent renoprotective effects against RI/R-induced renal injury, as compared with each monotherapy. Although co-treatment with ESC and EPO showed comparable antioxidant, anti-inflammatory and anti-apoptotic activities, and improvement in endothelial dysfunction, yet the underlying mechanisms were different. ESC exerted its nephroprotective activities mainly through inhibition of P2X7Rs that resulted in the preservation of renal tubular epithelial cells and substantial downregulation in NLRP3 inflammasome activation, whereas EPO acted mainly through activation of PI3K/Akt pathway. Our results provide a promising therapeutic approach to enhance the recovery of RI/R injury, however further studies are required to evaluate the beneficial therapeutic effects of combined ESC and EPO therapy for post-operative RI/R.

## Material and methods

### Plant material

*Vachellia farnesiana* flowers were collected from Al-Qaliobia Governorate, Egypt during March 2020. The identification and authentication of the collected plant was performed by Dr. Terase Labib, Department of Flora and Taxonomy, El-Orman Botanical Garden, Giza, Egypt. A voucher specimen (No. V.f/fl/2020) is kept in the herbarium of the garden. The experimental research and field studies on plants, including the collection of plant material, were approved by the Research Ethics Committee of TBRI and complied with the relevant institutional, national, and international guidelines and legislation.

### Extraction and fractionation

Fresh flowers of *V. farnesiana* (1.350 kg) were soaked for 3 days in 85% methanol (5 × 2 l) at room temperature. The combined extracts were filtered and evaporated under vacuum using Rotatory evaporator to obtain 85% methanol extract (132.85 g). The dried aqueous methanol extract was defatted via using petroleum ether (60–80 °C). Then, the defatted methanol extract undergone successive fractionation using organic solvents (e.g., dichloromethane, ethyl acetate, and *n*-butanol) in order to obtain 17.36, 15.85, 21.43, 43.74, and 31.18 g for petroleum ether, dichloromethane, ethyl acetate, *n*-butanol, and water extracts, respectively.

### Chromatographic isolation and purification of ESC compound

The chemical constituents of the tested extract were tentatively identified using a Thermo Finnigan (Thermo electron Corporation, OK, USA), coupled with an LCQ Duo ion trap mass spectrometer with an ESI source in negative ionization mode (ThermoQuest Corporation, Austin, TX, USA)^54^. The ethyl acetate extract (20.0 g) was consequently fractionated via using column chromatography (5 × 60 cm) packed with polyamide 6S as a stationary phase and eluted via a gradient mix elution system (Water: MeOH). A total of five major sub-fractions (F1–F5) were obtained. The obtained sub-fractions (F1–F4) were separately subjected to further extra purification using a multiple Sephadex LH-20 sub-columns (2 × 30 cm) eluted with (Water: MeOH with MeOH gradient) to obtain gallic acid, methyl gallate, *p*-coumaric acid, quercetin, taxifolin, naringenin, and quercetin 3-*O*-glucoside. While F5 (5.2 g) eluted by 85% MeOH from the main polyamide column was subjected to multiple Sephadex LH-20 sub-columns for extra purification eluted with methanol:water in gradient mix elution system to obtain ESC.

### Animals

Forty male Sprague–Dawley rats weighing 280–300 g were kept in the animal house of Theodor Bilharz Research Institute (TBRI), Egypt. Rats were maintained in individual polypropylene cages at 22–25 °C, 12–12 h dark and light cycle, and free access to standard laboratory chow diet and water. All procedures were conducted in adherence to the Guide for the Care and Use of Laboratory Animals (Eighth edition) of the National Institutes of Health, complied with the ARRIVE guidelines, and approved by the Research Ethics Committee of TBRI for the conduct of animal experiments (PT: 584; 22/2/2021).

### Surgical procedures

Each rat was anesthetized with 6% of Desflurane (Desflurane®, Baxter, UK) in 100% v/v oxygen via inhalation. Rats were placed in a supine position, the skin of the abdomen was shaved after disinfection with 70% alcohol, and a midline laparotomy incision was made. Subsequently, both kidneys with their pedicles were gently exposed and a non-traumatic vascular clamps (Bulldog clamps) were bilaterally placed on both the artery and vein of each kidney. After 45 min, successful reperfusion was obtained by removing the clamps, restoring the blood supply. The surgical incisions were stitched and covered with sterile gauze. After recovery from anesthesia, rats were returned to their sterile cages, for 24 h with free access to food and water. An identical operation was performed for the sham group without clamping the bilateral kidney pedicles.

### Experimental design

Rats were randomly divided into five groups of eight rats in each group. The animal groups were as follows; Sham (received vehicle alone), vehicle + RI/R, ESC + RI/R, EPO + RI/R, and ESC + EPO + RI/R. Rats were pretreated with ESC (50 mg/kg^11^) or vehicle (0.5% carboxymethyl cellulose in phosphate buffer saline (PBS), Sigma Aldrich, USA) via oral gavage for 7 days and 30 min prior to RI/R. Meanwhile, EPO (Eprex®, Janssen-Cialg, Switzerland) was administered in a single dose of 1000 U/kg, IP, 30 min^20^ prior to RI/R. Twenty-four hours following reperfusion, rats were sacrificed under light anesthesia using isoflurane (Forane®, Baxter, UK) inhalation, then blood and renal tissues were harvested and processed for subsequent biochemical, RT-PCR, histopathological, and immunohistochemical (IHC) examinations.

### Biochemical analyses

Collected blood samples were centrifuged at 3000×*g* for 10 min, then sera were separated and stored at − 80 °C for the spectrophotometric assessment of creatinine and urea levels (Biodiagnostics, Egypt), meanwhile the levels of Neutrophil gelatinase-associated lipocalin (NGAL) were determined using the commercial enzyme-linked immunosorbent assay (ELISA) kit (MyBiosource San Diego, USA), according to the manufacturer's instructions.

Moreover, part of the right kidney was homogenized in ice cold PBS and supernatants were used for the measurement of the malondialdehyde (MDA), glutathione peroxidase (GPx), and superoxide dismutase (SOD) levels using specific assay kits, as described by the manufacturer (Biodiagnostics, Egypt). Additionally, levels of tumor necrosis factor (TNF)-α, interleukin (IL)-1β, NF-κB p65, endothelial nitric oxide synthase (eNOS), p-PI3K, p-Akt, bax, and bcl2 were quantified using specific commercial ELISA kits as per manufacturer’s instructions (MyBiosource San Diego, USA).

### Quantitative real‑time polymerase chain reaction (qRT‑PCR).

Total RNA was isolated from the homogenized renal tissues with Easy-spin RNA extraction kit (Intron biotechnology, Korea). RNA was then converted into its complementary DNA (cDNA) RevertAid First Strand cDNA Synthesis Kit (Thermoscientific, USA). qRT-PCR was performed with StepOne™ Real-Time PCR (Applied Biosystems, USA) using Maxima SYBR Green qPCR Master Mix, no ROX (2×) (Thermoscientific, USA), according to the manufacturer’s instructions. Reverse transcription reaction conditions were processed at 42 °C for 15 min, followed by 3 min at 95 °C. The thermal cycling conditions were processed at 95 °C pre-denaturation, followed by 40 cycles (95 °C for 15 s; 60 °C for 30 s; 72 °C for 60 s). The following oligonucleotide primers were used: 5′-GTGGAGATCCTAGGTTTCTCTG-3′ (sense), 5′-CAGGATCTCATTCTCTTGGATC-3′ (antisense) for NLRP3, 5′-TTCTTCCCCTACATCCTGCT-3′ (sense), 5′-CTGTCAGAAGTCTTGTGCTCTG-3′ (antisense) for Caspase-1, 5′-CTACTCTTCGGTGGGGGCTT-3′ (sense), 5′-CTCTGGATCCGGGTGACTTT-3′ (antisense) for P2X7R, 5′-ACACCACGGTTTGGACTATGG-3′ (sense), 5′-GGCTACAGTAGTGGGCTTGG-3′ (antisense) for PI3K, 5′-ATGTCCGAGATCCTACCCTACG-3′ (sense), 5′-AGCGAAGAAGGAGTTGGTGTC-3′ (antisense) for Akt and 5′-TGATACAAAGACGGGACATCG-3′ (sense) and 5′-CACGATTTCCCTCTCAGC-3′ (antisense) for β-actin as an internal control for the normalization of target genes according to 2^−ΔΔCt^ method^55^. All the qRT-PCR expression experiments were performed in triplicates to ensure the reproducibility of obtained results.

### Histological examination

The kidneys were examined grossly for cortico-medullary demarcation, hemorrhage, and necrosis. Then, samples from left kidney were divided into two parts, one for the histopathology and the other for electron microscopy examinations. Briefly, kidney tissues were immediately fixed in a 10% formalin solution, processed, and embedded in paraffin. Sections of 4 microns thickness were then stained with: (1) hematoxylin and eosin (H&E) stain for histological evaluation of parenchymal injury (tubular injury necrosis, interstitial inflammatory infiltrate, and glomerular changes, (2) periodic acid-Schiff (PAS) reagent to highlight the capillary and tubular basement membranes, and (3) Masson's trichrome (MTC) staining to assess the fibrosis and highlight fibrinoid necrosis and fibrin thrombosis. At least 10 cortico-medullary fields were examined in each section at different magnification, and a semiquantitative analysis of tubulointerstitial injury was performed. The total tubular injury was graded on a scale of 0–4 based on the percentage of normal tubules and the extent of injury as followed: 0, absent; 1 (Minimal, 6–10%); 2 (Focal, 11–25%); 3 (Moderate, 26–50%); 4 (Diffuse, > 50%).

### Transmission electron microscope (TEM)

Kidney specimens for TEM were fixed in 2.5% glutaraldehyde in cacodylate buffer for 2 h at 4 °C. Tissues were then washed twice for 1 h each in cacodylate-sucrose buffer and post-fixed for 1 h at 4 °C in 2% osmium tetroxide. After dehydration in ascending graded ethanol, the samples were impregnated in Epon 812 substitute (EMBed-812 Kit, Electron Microscopy Science, USA) at room temperature, and polymerized at 60 °C for 48 h. Semi-thin sections were cut, stained with methylene blue-azure II, and examined by light microscopy to choose the region of interest for ultrathin sectioning. The ultrathin sections at 70 A° thickness were then prepared using an Ultracut R ultramicrotome (Leica, Vienna, Austria), and double stained with uranyl acetate and lead citrate. Ultrastructural examinations of the glomerular capillary loops, basement membrane, podocytes and signs of tubular injury were performed at 80 kV with Philips EM 208 S electron microscope (Philips Optics, Eindhoven, The Netherlands) provided by Electron Microscopy Department at TBRI.

### Immunohistochemical examinations of caspase-3 activity

Sections (5-microns thick) of formalin-fixed and paraffin-embedded tissue samples were prepared on charged glass slides and deparaffinized, hydrated, then treated for antigen retrieval at a high pH (pH = 8) using an automated immunostainer (Sigma, Aldrich, USA). Rabbit polyclonal anti-caspase antibodies (Sigma, Aldrich, USA); at dilution 1:200 was used to detect apoptosis. Goat anti-rabbit biotinylated immunoglobulins/HRP (Sigma, Aldrich, USA) was used at dilution 1:300. Streptavidin–biotin–peroxidase complex and peroxidase-DAB (3,3′-diaminobenzidine) (Sigma, Aldrich, USA) detection method was preformed according to the manufacturer’s instructions. Sections were counterstained with Mayer’s hematoxylin. Positive and negative control slides were included in each run. As a negative control, a tissue section was processed as described but with the omission of primary antibody.

### Statistical analysis

All data are expressed as mean ± SD. Statistical analyses were conducted using one-way analysis of variance (ANOVA) followed by Tukey–Kramer multiple comparison post hoc test for parametric analysis, whereas Kruskal–Wallis test followed by Dunn's post hoc was used for non-parametric analysis. Statistical differences were evaluated using GraphPad Prism software (USA, version 5.03). Statistical significance was set at *p* < 0.05, *p* < 0.01, and *p* < 0.001.

## Supplementary Information

Supplementary Information.

## Data Availability

All data generated or analyzed during this study are included in this published article and its supplementary information files.
