# Supplementary material for: Co-treatment with Esculin and erythropoietin protects against renal ischemia–reperfusion injury via P2X7 receptor inhibition and PI3K/Akt activation
Source: Sci Rep. 2022 Apr 14;12:6239. doi: 10.1038/s41598-022-09970-8 (PMC9010483; doi:10.1038/s41598-022-09970-8)
Supplement: Supplementary file 1 — Supplementary Information. [file 41598_2022_9970_MOESM1_ESM.docx]

**Co-treatment with Esculin and erythropoietin protects against renal ischemia-reperfusion injury via P2X7 receptor inhibition and PI3K/Akt activation**

Walaa H. El-Maadawy^a^, Marwa Hassan^b^, Ehab Hafiz^c^, Mohamed H. Badawy ^d^, Samir Eldahshan^d^, AbdulRahman AbuSeada^e^, Maha A.M. El-Shazly^f^, Mosad A. Ghareeb^f^

^a^Pharmacology Department, Theodor Bilharz Research Institute, Warrak El-Hadar, Imbaba (P.O. 30), Giza 12411, Egypt.

^b^Immunology Department, Theodor Bilharz Research Institute, Warrak El-Hadar, Imbaba (P.O. 30), Giza 12411, Egypt.

^c^Electron Microscopy Department, Theodor Bilharz Research Institute, Warrak El-Hadar, Imbaba (P.O. 30), Giza 12411, Egypt.

^d^Urology Department, Theodor Bilharz Research Institute, Warrak El-Hadar, Imbaba (P.O. 30), Giza 12411, Egypt.

^e^Anesthesia Department, Theodor Bilharz Research Institute, Warrak El-Hadar, Imbaba (P.O. 30), Giza 12411, Egypt.

^f^Medicinal Chemistry Department, Theodor Bilharz Research Institute, Warrak El-Hadar, Imbaba (P.O. 30), Giza 12411, Egypt.

**Corresponding author:**

Walaa H. El-Maadawy, Pharmacology department, Theodor Bilharz Research Institute, Kornaish El Nile, Warrak El-Hadar, Imbaba (P.O. 30), Giza 12411, Egypt. Email: [w.elmadawy@tbri.gov.eg](mailto:w.elmadawy@tbri.gov.eg)

~~Mosad A. Ghareeb, Medicinal Chemistry Department, Theodor Bilharz Research Institute, Warrak El-Hadar, Imbaba (P.O. 30), Giza 12411, Egypt. Email:~~ [~~m.ghareeb@tbri.gov.eg~~](mailto:m.ghareeb@tbri.gov.eg)

1. **Materials and Methods**
   1. *Chemicals and Reagents*

2,2′-diphenyl-1-picrylhydrazyl (DPPH) free radical, Folin-Ciocalteu’s reagent, gallic acid, ascorbic acid, and rutin were purchased from Sigma-Aldrich Company (Steinheim, Germany). Sodium carbonate, ammonium molybdate, sodium phosphate, methanol, petroleum ether (60-80 °C), dichloromethane, ethyl acetate and *n*-butanol were obtained from El-Nasr Pharmaceutical Chemicals Company (Cairo, Egypt). All spectrophotometric estimations were performed on Spectronic 601 UV-Vis Spectrophotometer (Milton Roy, USA). All other solvents and reagents were of the highest commercially available grade.

- 1. *Determination of total phenolic content (TPC)*

The total phenolic content (TPC) was estimated via using Folin-Ciocalteu’s reagent according to the reported procedures [1].

- 1. *Determination of Total Flavonoid Content (TFC)*

The content of flavonoid content (TFC) was estimated according to the reported procedures [2].

- 1. *Determination of Total Antioxidant Capacity (TAC)*

Total antioxidant capacity (TAC) of each tested extract was evaluated according to phosphomolybdenum assay [3].

- 1. *Determination of free radical scavenging activity*

Free radical scavenging activity was evaluated using DPPH assay according to the reported procedures [4].

- 1. *Statistical analysis*

All data were presented as mean ± S.D. using SPSS 13.0 program (SPSS Inc. USA).

1. **Results and Discussion**
   1. *Total phenolic & flavonoids contents and antioxidant activity*

In TPC assay, the phenolic contents for the tested extracts ranged from 84.44 to 215.55 mg GAE/g dry extract. The results are in the order: EtOAc ˃ *n*-BuOH ˃ 85%MeOH **(Table 1S)**. Similarly, in TFC assay, the flavonoid contents for the tested extracts ranged from 206.60 to 384.15 mg RE/g dry extract. Also, the results are in the order: EtOAc ˃ *n*-BuOH ˃ 85%MeOH **(Table 1S)**. The antioxidant activity was evaluated via two assays (DPPH radical, and phosphomolybdenum). In DPPH assay, the IC_50_ values for the tested extracts ranged from 37.31 to 117.28 µg/ ml compared to ascorbic acid with IC_50_ equal to7.50 µg/ml. The ethyl acetate extract was the most potent with IC_50_ value of 37.31, followed by *n*-BuOH extract with IC_50_ value of 71.41, and the weakest activity was recorded by 85% MeOH with IC_50_ value of 117.28 µg/ml **(Table 1S)**. Furthermore, in phosphomolybdenum assay the TAC values are 371.31, 293.29, and 162.66 mg AAE/g dry extract, respectively for EtOAc, *n*-BuOH and 85% MeOH extracts **(Table 1S)**. Ramli et al. (2011) reported that the EC_50_ value of the ethanolic leaf extract of *A. farnesiana* grown in Bangkok was equal to 56 μg/ ml, and it's free radical scavenging activity returned to the presence of polyphenolic compounds like; quercetin-galloylglucoside, rutin, quercetin-pentoside, quercetin-deoxyhexoside, and 5-diosmetin diglycoside [5]. The crude extract from *A. farnesiana* pods grown in Mexico showed DPPH scavenging percent (%) of 95.18 and TPC value of 76 GAE g/dry extract [6]. Previous reports revealed that the antioxidant activity of *V. farnesiana* mainly based on their polyphenolic contents [7].

**Table 1S:** Total phenolic content (TPC), total flavonoid content (TFC), total antioxidant capacity (TAC) and free radical scavenging activity (DPPH) values of 85% methanol extract of *V. farnesiana* flowers and its derived fractions

| **Sample** | **Total phenolic content**  **(mg GAE/g dry extract)^1,2^** | **Total flavonoid content**  **(mg RE/g dry extract)^3^** | **Total antioxidant capacity**  **(mg AAE/g dry extract)^4^** | **DPPH**  **(IC_50_ µg/ml)^5^** |
| --- | --- | --- | --- | --- |
| 85% MeOH | 84.44 ± 1.18 | 206.60 ± 1.14 | 162.66 ± 2.30 | 117.28 ± 0. 04 |
| EtOAc | 215.55 ± 3.85 | 384.15 ± 1.98 | 371.31 ± 3.42 | 37.31 ± 0.75 |
| *n*-BuOH | 142.22 ± 3.84 | 323.19 ± 2.31 | 293.29 ± 2.21 | 71.41 ± 0.35 |
| Ascorbic acid | - | - | - | 7.50 ± 1.50 |

^1^Results are (means ± S.D.) (n = 3); ^2^GAE (Gallic acid equivalent); ^3^RE (Rutin acid equivalent); ^4^AAE (Ascorbic acid equivalent); ^5^IC_50_: The amount of extract needed to scavenge 50% of DPPH radicals.

- 1. *Chemical characterization of V. farnesiana flowers ethyl acetate extract using LC-ESI-MS/MS*

LC-ESI-MS/MS analysis led to identification of 48 compounds in the EtOAc extract of *V. farnesiana* flowers using negative ion mode **(Table 2S).** The identification based on retention times, fragmentation patterns and reported data.

*Phenolic & organic acids and their derivatives*

A molecular ion peak was detected at R_t_ (0.77 min) with a deprotonated ion [M-H]^−^ at *m/z* 133, and daughter ion was detected at *m/z* 115 [M-H-18]^−^ due to the neutral loss of H_2_O moiety. This fragmentation pattern was typically assigned to malic acid [8]. A molecular ion peak was detected at R_t_ (1.0), it showed a deprotonated ion [M-H]^−^ at *m/z* 169 and a diagnostic fragment ion was detected at *m/z* 125 corresponding to the neutral loss of CO_2_ moiety [M-H-44]^−^. In this regard, the compound was tentatively identified as gallic acid [9]. Two molecular ion peak were detected at R_t_ (2.13 and 2.20 min) with deprotonated ions [M-H]^−^ at *m/z* 183 and MS^n^ ions at *m/z* 169, and 125. These compounds could be identified as methyl gallate [10]. Two molecular ion peak were detected at R_t_ (2.61 and 2.66 min) with deprotonated ions [M-H]^−^ at *m/z* 315 and MS^n^ ions at *m/z* 300, 272, 244, and 200. These compounds could be identified as Methylellagic acid [11]. A molecular ion peak was detected at R_t_ (4.71 min) with a deprotonated ion [M-H]^−^ at *m/z* 163, and daughter ion was detected at *m/z* 119 [M-H-44]^−^ due to the neutral loss of CO_2_ moiety. This fragmentation pattern was typically assigned to *p*-Coumaric acid **[**12]. A molecular ion peak was detected at R_t_ (5.02 min) with a deprotonated ion [M-H]^−^ at *m/z* 167, and daughter ions were detected at *m/z* 152 [M-H-CH_3_]^−^, 123 [M-H-CO_2_]^−^, and 108[M-H-CO_2_-CH_3_]^−^. This fragmentation pattern was typically assigned to Vanilic acid [12]. A molecular ion peak was detected at R_t_ (5.61 min) with a deprotonated ion [M-H]^−^ at *m/z* 509, and a daughter ion was detected at *m/z* 301 [M-H-208]^−^ corresponding to ellagic acid moiety, in addition to characteristic fragments of ellagic acid were observed at *m/z* 284, 257, 229, and 185. This fragmentation pattern was typically assigned to Ellagic acid derivatives [13]. A molecular ion peak was detected at R_t_ (6.58 min) with a deprotonated ion [M-H]^−^ at *m/z* 151, and daughter ion were detected at *m/z* 107, and 93. This fragmentation pattern was typically assigned to *O*-Homosalicylic acid [14]. A molecular ion peak was detected at R_t_ (6.67 min) with a deprotonated ion [M-H]^−^ at *m/z* 187, and daughter ion were detected at *m/z* 169, and 125. This fragmentation pattern was typically assigned to Gallic acid monohydrate [15]. A molecular ion peak was detected at R_t_ (7.10 min) with a deprotonated ion [M-H]^−^ at *m/z* 207, and a daughter ion was detected at *m/z* 163. This compound could be identified as 2,5-Dimethoxycinnamic acid  [16]. A molecular ion peak was detected at R_t_ (7.94 min) with a deprotonated ion [M-H]^−^ at *m/z* 177, and daughter ions were detected at *m/z* 145 and 118. This compound could be identified as *p*-Coumaric acid methyl ester [17]. A molecular ion peak was detected at R_t_ (8.15 min) with a deprotonated ion [M-H]^−^ at *m/z* 447, and daughter ions were detected at *m/z* 301, 257, 229, and 185. This compound could be identified as Ellagic acid deoxy­hexoside [18]. A molecular ion peak was detected at R_t_ (9.25 min) with a deprotonated ion [M-H]^−^ at *m/z* 329, and daughter ions were detected at *m/z* 167, 152, 123, and 108. This compound could be identified as 1-*O*-vanilloyl-beta-D-glucose [19]. A molecular ion peak was detected at R_t_ (10.08 min) with a deprotonated ion [M-H]^−^ at *m/z* 385, and daughter ions were detected at *m/z* 223, and 179. This compound could be identified as Sinapoyl D-glucoside [20]. A molecular ion peak was detected at R_t_ (10.18 min) with a deprotonated ion [M-H]^−^ at *m/z* 341, and daughter ions were detected at *m/z* 179, and 161. This compound could be identified as Caffeoyl glucoside [21]. A molecular ion peak was detected at R_t_ (10.41 min) with a deprotonated ion [M-H]^−^ at *m/z* 341, and a daughter ion was detected at *m/z* 191. This compound could be identified as 5-Hydroxyferulic acid [12]. A molecular ion peak was detected at R_t_ (11.02 min) with a deprotonated ion [M-H]^−^ at *m/z* 311, and daughter ions were detected at *m/z* 149, and 131. This compound could be identified as p-coumaryl alcohol 4-*O*-β-D-glucoside [22]. A molecular ion peak was detected at R_t_ (13.60 min) with a deprotonated ion [M-H]^−^ at *m/z* 721, and daughter ions were detected at *m/z* 191, and 163. This compound could be identified as Dihydroxyferuloyl-p coumaroylquinic acid [16]. A molecular ion peak was detected at R_t_ (14.80 min) with a deprotonated ion [M-H]^−^ at *m/z* 459, and daughter ions were detected at *m/z* 415, 295, 253, 239, 225, and 166. This compound could be identified as Chrysophanol-sinapoyl [23]. A molecular ion peak was detected at R_t_ (14.99 min) with a deprotonated ion [M-H]^−^ at *m/z* 559, and daughter ions were detected at *m/z* 381, 335, 223, 191, 176, 135, and 104. This compound could be identified as 1-*O*-caffeoyl-3-*O*-sinapoylquinic acid [24].

*Coumarin derivatives*

A molecular ion peak was detected at R_t_ (1.45 min) with a deprotonated ion [M-H]^−^ at *m/z* 339, and a daughter ion was detected at *m/z* 177 [M-H-162]^−^ due to the neutral loss of glucose moiety. This fragmentation pattern was typically assigned to Esculin [25]. A molecular ion peak was detected at R_t_ (11.22 min) with a deprotonated ion [M-H]^−^ at *m/z* 201, and daughter ions were detected at *m/z* 145, 129, and 117. This fragmentation pattern was typically assigned to Bergaptol [26].

*Flavonoids and their derivatives*

A molecular ion peak was detected at R_t_ (3.30 min) with a deprotonated ion [M-H]^−^ at *m/z* 345, and daughter ions were detected at *m/z* 330, 328, 315, 314, 313, 300, 299, and 285. This fragmentation pattern was typically assigned to Syringetin [14]. A molecular ion peak was detected at R_t_ (3.43 min) with a deprotonated ion [M-H]^−^ at *m/z* 281, and daughter ions were detected at *m/z* 266, 261, 251, 238, 237, and 233. This fragmentation pattern was typically assigned to Syringetin [14]. A molecular ion peak was detected at R_t_ (6.77 min) with a deprotonated ion [M-H]^−^ at *m/z* 415, and daughter ions were detected at *m/z* 313, 253, 235, 203, 193, and 109. This fragmentation pattern was typically assigned to Daidzein 7-*O*-β-D-glucoside [14]. A molecular ion peak was detected at R_t_ (6.90 min) with a deprotonated ion [M-H]^−^ at *m/z* 447, and daughter ions were detected at *m/z* 285, 267, 257, 243, 241, 217, 199, 175, and 151. This fragmentation pattern was typically assigned to Luteolin 7-*O*-glucoside [27]. A molecular ion peak was detected at R_t_ (7.59 min) with a deprotonated ion [M-H]^−^ at *m/z* 301, and daughter ions were detected at *m/z* 271, 269, 255, 229, 179, 169, and 151. This fragmentation pattern was typically assigned to Quercetin [28]. A molecular ion peak was detected at R_t_ (7.69 min) with a deprotonated ion [M-H]^−^ at *m/z* 461, and daughter ions were detected at *m/z* 341, 312, 241, 215, and 136. This fragmentation pattern was typically assigned to Diosmetin-8-C-β-D-glucoside [29]. A molecular ion peak was detected at R_t_ (8.39 min) with a deprotonated ion [M-H]^−^ at *m/z* 271, and daughter ions were detected at *m/z* 177, 151, 119, and 107. This fragmentation pattern was typically assigned to Naringenin [30]. A molecular ion peak was detected at R_t_ (8.55 min) with a deprotonated ion [M-H]^−^ at *m/z* 299, and daughter ions were detected at *m/z* 179, and 151. This fragmentation pattern was typically assigned to Diosmetin [31]. A molecular ion peak was detected at R_t_ (8.86 min) with a deprotonated ion [M-H]^−^ at *m/z* 303, and daughter ions were detected at *m/z* 285, 275, 241, 217, 199, 175, and 151. This fragmentation pattern was typically assigned to Taxifolin [32]. A molecular ion peak was detected at R_t_ (9.54 min) with a deprotonated ion [M-H]^−^ at *m/z* 287, and daughter ions were detected at *m/z* 269, 259, 243, 215, 201, 151, and 125. This fragmentation pattern was typically assigned to Dihydrokaempferol [24]. A molecular ion peak was detected at R_t_ (12.25 min) with a deprotonated ion [M-H]^−^ at *m/z* 463, and daughter ions were detected at *m/z* 301, 273, 257, 255, 229, 179, and 151. This fragmentation pattern was typically assigned to Quercetin 3-glucoside [27]. Two molecular ion peaks were detected at R_t_ (13.88 & 14.12 min) with deprotonated ions [M-H]^−^ at *m/z* 533, and daughter ions were detected at *m/z* 447, and 285. This fragmentation pattern was typically assigned to Kaempferol 3-(6''-malonylglucoside) [18]. Two molecular ion peaks were detected at R_t_ (15.49 & 15.55 min) with deprotonated ions [M-H]^−^ at *m/z* 445, and daughter ions were detected at *m/z* 269, 227, 225, 201, 197, 181, and 175. This fragmentation pattern was typically assigned to Apigenin 7-*O*-glucuronide [27]. A molecular ion peak was detected at R_t_ (15.79 min) with a deprotonated ion [M-H]^−^ at *m/z* 461, and daughter ions were detected at *m/z* 286, 285, 151, and 133. This fragmentation pattern was typically assigned to Luteolin 4'-*O*-glucuronide [33]. Four molecular ion peaks were detected at R_t_ (16.73, 16.90, 17.06 & 17.26 min) with deprotonated ions [M-H]^−^ at *m/z* 447, and daughter ions were detected at *m/z* 285, 267, 257, 243, 241, 217, and 199. This fragmentation pattern was typically assigned to Luteolin 7-*O*-glucoside [27].

*Naphthalenedione derivatives*

A molecular ion peak was detected at R_t_ (7.49 min) with a deprotonated ion [M-H]^−^ at *m/z* 263, and daughter ions were detected at *m/z* 245, 235, 219, 207, and 191. This fragmentation pattern was typically assigned to Spinochrome A [34].

*Iridoids*

Two molecular ion peaks were detected at R_t_ (9.68 & 9.80 min) with deprotonated ions [M-H]^−^ at *m/z* 509, and daughter ions were detected at *m/z* 347, 277, 233, 165, and 121. This fragmentation pattern was typically assigned to Demethyl ligstroside [35]. A molecular ion peak was detected at R_t_ (9.80 min) with a deprotonated ion [M-H]^−^ at *m/z* 523, and daughter ions were detected at *m/z* 291, and 259. This fragmentation pattern was typically assigned to Ligstroside [35].

*Other nuclei*

A molecular ion peak was detected at R_t_ (8.68 min) with a deprotonated ion [M-H]^−^ at *m/z* 327, and daughter ions were detected at *m/z* 228 and 212. This fragmentation pattern was typically assigned to Selinidin [26] .

**Table 2S:** Tentative identification of polyphenolic constituents in the ethyl acetate extract of *V. farnesiana* flowers using LC-ESI-MS/MS

| **References** | **Proposed compound** | **MS^2^ fragments (*m/z*)** | **M.F.** | **M.wt.** | ***m/z***  **[M-H]^-^** | **R_t_ (min)** | **No.** |
| --- | --- | --- | --- | --- | --- | --- | --- |
| [8] | Malic acid | 115 | C_4_H_6_O_5_ | 134 | 133 | 0.77 | 1 |
| [9] | Gallic acid | 125 | C_7_H_6_O_5_ | 170 | 169 | 1.0 | 2 |
| [25] | Esculin | 177 | C_15_H_16_O_9_ | 340 | 339 | 1.45 | 3 |
| [10] | Methyl gallate | 183, 169, 125 | C_8_H_8_O_5_ | 184 | 183 | 2.13 | 4 |
| [10] | Methyl gallate | 183, 169, 125 | C_8_H_8_O_5_ | 184 | 183 | 2.20 | 5 |
| [11] | Methylellagic acid | 300, 272, 244, 200 | C_15_H_8_O_8_ | 316 | 315 | 2.61 | 6 |
| [11] | Methylellagic acid | 300, 272, 244, 200 | C_15_H_8_O_8_ | 316 | 315 | 2.66 | 7 |
| [14] | Syringetin | 330, 328, 315, 314, 313, 300, 299, 285 | C_17_H_14_O_8_ | 346 | 345 | 3.30 | 8 |
| [14] | Chrysin 5,7-dimethyl ether | 266, 261, 251, 238, 237, 233 | C_17_H_14_O_4_ | 282 | 281 | 3.43 | 9 |
| [12] | *p*-Coumaric acid | 163, 119 | C_9_H_8_O_3_ | 164 | 163 | 4.71 | 10 |
| [12] | Vanilic acid | 152, 123, 108 | C_8_H_8_O_4_ | 168 | 167 | 5.02 | 11 |
| [13] | Ellagic acid derivatives | 509, 301, 284, 257, 229, 185 | C_24_H_14_O_13_ | 510 | 509 | 5.61 | 12 |
| [14] | *O*-Homosalicylic acid | 151, 107, 93 | C_8_H_8_O_3_ | 152 | 151 | 6.58 | 13 |
| [15] | Gallic acid monohydrate | 187, 169, 125 | C_7_H_8_O_6_ | 188 | 187 | 6.67 | 14 |
| [14] | Daidzein 7-O-β-D-glucoside | 415, 313, 253, 235, 203, 193, 109 | C_21_H_20_O_9_ | 416 | 415 | 6.77 | 15 |
| [27] | Luteolin 7-*O*-glucoside | 447, 285, 267, 257, 243, 241, 217, 199, 175 | C_21_H_20_O_11_ | 448 | 447 | 6.90 | 16 |
| [16] | 2,5-Dimethoxycinnamic acid | 207, 163 | C_11_H_12_O_4_ | 208 | 207 | 7.10 | 17 |
| [33] | Spinochrome A | 245, 235, 219, 207, 191 | C_12_H_8_O_7_ | 264 | 263 | 7.49 | 18 |
| [28] | Quercetin | 271, 269, 255, 229, 179, 169, 151 | C_15_H_10_O_7_ | 302 | 301 | 7.59 | 19 |
| [29] | Diosmetin -8-C-β-D-glucoside | 341, 312, 241, 215, 136 | C_22_H_22_O_11_ | 462 | 461 | 7.69 | 20 |
| [17] | p-Coumaric acid methyl ester | 145, 118 | C_10_H_9_O_3_ | 178 | 177 | 7.94 | 21 |
| - | Unknown | - | - | 763 | 762 | 8.02 | 22 |
| [18] | Ellagic acid deoxy­hexoside | 301, 257, 229, 185 | C_20_H_16_O_12_ | 448 | 447 | 8.15 | 23 |
| [30] | Naringenin | 177, 151, 119, 107 | C_15_H_12_O_5_ | 272 | 271 | 8.39 | 24 |
| [31] | Diosmetin | 179, 151 | C_16_H_12_O_6_ | 300 | 299 | 8.55 | 25 |
| [26] | Selinidin | 228, 212 | C_19_H_20_O_5_ | 328 | 327 | 8.68 | 26 |

***Continue* Table 2S:**

| **References** | **Proposed compound** | **MS^2^ fragments (*m/z*)** | **M.F.** | **M.wt.** | ***m/z***  **[M-H]^-^** | **R_t_ (min)** | **No.** |
| --- | --- | --- | --- | --- | --- | --- | --- |
| [32] | Taxifolin | 303, 285, 275, 241, 217, 199, 175, 151 | C_15_H_12_O_7_ | 304 | 303 | 8.86 | 27 |
| [19] | 1-*O*-vanilloyl-beta-D-glucose | 167, 152, 123, 108 | C_14_H_18_O_9_ | 330 | 329 | 9.25 | 28 |
| [24] | Dihydrokaempferol | 287, 269, 259, 243, 215, 201, 151, 125 | C_15_H_12_O_6_ | 288 | 287 | 9.54 | 29 |
| [35] | Demethyl ligstroside | 509, 347, 277, 233, 165, 121 | C_24_H_30_O_12_ | 510 | 509 | 9.68 | 30 |
| [35] | Demethyl ligstroside | 509, 347, 277, 233, 165, 121 | C_24_H_30_O_12_ | 510 | 509 | 9.80 | 31 |
| [35] | Ligstroside | 291, 259 | C_25_H_32_O_12_ | 524 | 523 | 9.80 | 31 |
| [20] | Sinapoyl D-glucoside | 223, 179 | C_17_H_22_O_10_ | 386 | 385 | 10.08 | 32 |
| [21] | Caffeoyl glucoside | 179, 161 | C_15_H_18_O_9_ | 342 | 341 | 10.18 | 33 |
| [12] | 5-Hydroxyferulic acid | 191 | C_10_H_10_O_5_ | 210 | 209 | 10.41 | 34 |
| [22] | *p*-coumaryl alcohol 4-*O*-β-D-glucoside | 149, 131 | C_15_H_20_O_7_ | 312 | 311 | 11.02 | 35 |
| [26] | Bergaptol | 145, 129, 117 | C_11_H_6_O_4_ | 202 | 201 | 11.22 | 36 |
| [27] | Quercetin 3-glucoside | 463, 301, 273, 257, 255, 229, 179, 151 | C_21_H_20_O_12_ | 464 | 463 | 12.25 | 37 |
| [16] | Dihydroxyferuloyl-p coumaroylquinic acid | 191, 163 | C_36_H_33_O_16_ | 722 | 721 | 13.60 | 38 |
| [18] | Kaempferol 3-(6''-malonylglucoside) | 447, 285 | C_24_H_22_O_14_ | 534 | 533 | 13.88 | 39 |
| [18] | Kaempferol 3-(6''-malonylglucoside) | 447, 285 | C_24_H_22_O_14_ | 534 | 533 | 14.12 | 40 |
| [23] | Chrysophanol-sinapoyl | 459, 415, 295, 253, 239, 225, 166 | **-** | 460 | 459 | 14.80 | 41 |
| [24] | 1-*O*-caffeoyl-3-*O*-sinapoylquinic acid | 381, 335, 223, 191, 176, 135, 104 | C_27_H_28_O_13_ | 560 | 559 | 14.99 | 42 |
| - | Unknown | - | - | 536 | 535 | 15.19 | 43 |
| [27] | Apigenin 7-*O*-glucuronide | 445, 269, 227, 225, 201, 197, 181, 175 | C_21_H_18_O_11_ | 446 | 445 | 15.49 | 44 |
| [27] | Apigenin 7-*O*-glucuronide | 445, 269, 227, 225, 201, 197, 181, 175 | C_21_H_18_O_11_ | 446 | 445 | 15.55 | 45 |
| [33] | Luteolin 4'-*O*-glucuronide | 461, 286, 285, 151, 133 | C_21_H_18_O_12_ | 462 | 461 | 15.79 | 46 |
| - | Unknown | - | - | 484 | 483 | 16.07 | 47 |
| [27] | Luteolin 7-*O*-glucoside | 447, 285, 267, 257, 243, 241, 217, 199 | C_21_H_20_O_11_ | 448 | 447 | 16.73 | 48 |
| [27] | Luteolin 7-*O*-glucoside | 447, 285, 267, 257, 243, 241, 217, 199 | C_21_H_20_O_11_ | 448 | 447 | 16.90 | 49 |
| [27] | Luteolin 7-*O*-glucoside | 447, 285, 267, 257, 243, 241, 217, 199 | C_21_H_20_O_11_ | 448 | 447 | 17.06 | 50 |
| [27] | Luteolin 7-*O*-glucoside | 447, 285, 267, 257, 243, 241, 217, 199 | C_21_H_20_O_11_ | 448 | 447 | 17.26 | 51 |

2.3. *Structural elucidation of the isolated compounds*

The structural elucidation of the isolated compounds based on physicochemical examination, matched LC-ESI-MS/MS peaks, acid hydrolysis and Co-chromatography. Gallic acid was isolated as off-white powder, m.p. 251-252ºC. It showed a violet spot under UV-light [9]. Methyl gallate was isolated as off-white fine crystals, m.p. 200-202ºC. It showed a deep violet spot under UV-light [9]. p-coumaric acid was isolated as white powder, m.p. 211-213ºC. It showed a blue spot under UV-light [36]. Quercetin was isolated as yellow amorphous powder, m.p. 305-307ºC. It showed a yellow spot under UV-light [37]. Taxifolin was isolated as a dark yellow amorphous powder, m.p. 234-236ºC. It showed a yellow spot under UV-light [38]. Naringenin was isolated as faint yellow powder, m.p. 252-254ºC. It showed fluorescence green-yellowish spot under UV-light [38]. Quercetin 3-*O*-glucoside was isolated as yellow powder, m.p. 227-229ºC. It showed dark purple spot under UV-light [39].

**Supplementary citations:**

[1] K.S. Kumar, K. Ganesan, P.V. [Subba](https://www.sciencedirect.com/science/article/abs/pii/S0308814607008102#!) Rao. Antioxidant potential of solvent extracts of *Kappaphycus alve*rezii (Doty). Edible seaweed. Food Chem. 107 (2008) 289-295. https://doi.org/[10.1016/j.foodchem.2007.08.016](https://doi.org/10.1016/j.foodchem.2007.08.016).

[2] A. Kumaran, R.J. Karunakaran. *In vitro* antioxidant activities of methanol extracts of five *Phyllanthus* species from India. LWT-Food Sci. Technol. 40 (2006) 344-352. https://doi.org/10.1016/j.lwt.2005.09.011.

[3] P. Prieto, M. Pineda, M. Aguilar. Spectrophotometric quantation of antioxidant capacity through the formation of a phosphomolybdenum complex: Specific application to the determination of vitamin E. Anal. Biochem. 269 (1999) 337- 341. https://doi.org/10.1006/abio.1999.4019.

[4] A. Shirwaikar, K. Rajendran, I.S. Punithaa. *In vitro* antioxidant studies on the benzyl tetra isoquinoline alkaloid berberine. Biol. Pharm. Bull. 29 (2006) 1906-1910. https://doi.org/10.1248/bpb.29.1906.

[5] S. Ramli, K. Harada, N. Ruangrungsi. Antioxidant, antimicrobial and cytotoxicity activities of *Acacia farnesiana* (L.) Willd. leaves ethanolic extract. Pharmacogn J. 3 (2011) 50-58. https://doi.org/10.5530/pj.2011.23.8.

[6] C.D. Puga, M. Cuchillo-Hilario, J.G.E. Mendoza, O.M. Campos, E.M. Jijón, M.D. Martínez, M.A.A. Izazaga, J.A.L. Solano, J.P. Chaverri. Antioxidant activity and protection against oxidative-induced damage of *Acacia shaffneri* and *Acacia farnesiana* pods extracts: *in vitro* and *in vivo* assays. BMC Complement Altern Med. 15 (2015) 435. https://doi.org/10.1186/s12906-015-0959-y.

[7] C.D. Puga, M. Cuchillo-Hilario, A.N. Ocaña, O.N. Medina-Campos, A.N. Camacho, T.R. Apan, Z.G. López-Tecpoyotl, M.D. Martínez, M.A. Álvarez-Izazaga, Y.R.C. Martínez, V. Sánchez-Quezada, F.E. Gómez, I. Torre-Villalvazo, J.F. Carballeda, M.R. Camacho-Corona, J. Pedraza-Chaverri. Phenolic compounds in organic and aqueous extracts from *Acacia farnesiana* pods analyzed by ULPS-ESI-Q-oa/TOF-MS. *In vitro* antioxidant activity and anti-Inflammatory response in CD-1 mice. Molecules. 23 (2018) 2386. https://doi.org/10.3390/molecules23092386.

[8] M. Sobeh, M.F. Mahmoud, R.A. Hasan, M.A.O. Abdelfattah, O.M. Sabry, M.A. Ghareeb, A.M. El-Shazly, M. Wink. Tannin-rich extracts from *Lannea stuhlmannii* and *Lannea humilis* (Anacardiaceae) exhibit hepatoprotective activities *in vivo* via enhancement of the anti-apoptotic protein Bcl-2. Sci Rep. 8 (2018) 9343. https://doi.org/10.1038/s41598-018-27452-8.

[9] M.A. Ghareeb, M Sobeh, W.H. El-Maadawy, [H.S. Mohammed](https://pubmed.ncbi.nlm.nih.gov/?term=Mohammed+HS&cauthor_id=31546777), H. [Khalil](https://pubmed.ncbi.nlm.nih.gov/?term=Khalil+H&cauthor_id=31546777), [S. Botros](https://pubmed.ncbi.nlm.nih.gov/?term=Botros+S&cauthor_id=31546777), [M. Wink](https://pubmed.ncbi.nlm.nih.gov/?term=Wink+M&cauthor_id=31546777). Chemical profiling of polyphenolics in *Eucalyptus globulus* and evaluation of its hepato–renal protective potential against cyclophosphamide induced toxicity in mice. Antioxidants. 8 (2019) 415. https://doi.org/10.3390/antiox8090415.

[10] M.A. Ghareeb, T. Mohamed, A.M. Saad, L.A. Refahy, M. Sobeh, M. Wink. HPLC-DAD-ESI-MS/MS analysis of fruits from *Firmiana simplex* (L.) and evaluation of their antioxidant and antigenotoxic properties*.* J. Pharm. Pharmacol. 70 (2018a) 133-142. https://doi.org/10.1111/jphp.12843.

[11] J. Sun, P. Chen. UHPLC/HRMS analysis of African Mango (*Irvingia gabonensis*) seeds, extract and related dietary supplements. J Agric Food Chem. 60 (2012) 8703-8709. https://doi.org/10.1021/jf302703u.

[12] M.A. Ghareeb, M. Sobeh, S. Rezq, A.M. El-Shazly, M.F. Mahmoud, M. Wink. HPLC-ESI-MS/MS profiling of polyphenolics of a leaf extract from *Alpinia zerumbet* (Zingiberaceae) and its anti-inflammatory, anti-nociceptive, and antipyretic activities *in vivo*. Molecules. 23 (2018b) 3238. https://doi.org/10.3390/molecules23123238.

[13] D. Fraternale, D. Ricci, G. Verardo, A. Gorassini, V. Stocchi, P. Sestili. Activity of *Vitis vinifera* tendrils extract against phytopathogenic fungi. Nat. Prod. Commun. 10 (2015) 1037-1042. https://doi.org/10.1177/1934578X1501000661.

[14] K.A. Reed. Identification of phenolic compounds from Peanut skin using HPLC-MS^n^. Ph.D. thesis, Faculty of the Virginia Polytechnic Institute and State University, Blacksburg, Virginia, 2009.

[15] J. Kang, W. Price, J. Ashton, L. Tapsell, S. Johnson. Identification and characterization of phenolic compounds in hydromethanolic extracts of sorghum wholegrains by LC-ESI-MS^n^. Food Chem. 211 (2016) 215-226. https://doi.org/10.1016/j.foodchem.2016.05.052.

[16] Á. Nagy, L. Abrankó. Profiling of hydroxycinnamoylquinic acids in plant extracts using in-source CID fragmentation. J. Mass Spectrom. 51 (2016) 1130-1145. https://doi.org/10.1002/jms.3847.

[17] D. Šuković, B. Knežević, U. Gašić, M. Sredojević, I. Ćirić, S. Todić, J. Mutić, Ž. Tešić. Phenolic profiles of leaves, grapes and wine of Grapevine variety vranac (*Vitis vinifera* L.) from Montenegro. Foods. 9 (2020) 138. https://doi.org/10.3390/foods9020138.

[18] M. Kajdžanoska, V. Gjamovski, M. Stefova. HPLC-DAD-ESI-MS^n^ identification of phenolic compounds in cultivated strawberries from Macedonia. Maced. J. Chem. Chem. Eng*.* 29 (2010) 18-194. https://doi.org/10.20450/mjcce.2010.165.

[19] S. Li, Z. Lin, H. Jiang, L. Tong, H. Wang, S. Chen. Rapid identification and assignation of the active ingredients in Fufang Banbianlian injection using HPLC-DAD-ESI-IT-TOF-MS. J. Chromatogr. Sci. 54 (2016) 1225-1237. https://doi.org/10.1093/chromsci/bmw055.

[20] R.F. Ahmed, E.A. Elkhrisy, W.A. El-Kashak, M.A. El Raey, M.I. Nassar, S.A. El- Aboutab. Structural characterization of polyphenolics in *Livistona chinensis using* HPLC-PDA-MS. J. Adv. Pharm. Res. 3 (2019): 23-29. https://doi.org/10.21608/APRH.2018.6527.1072.

[21] Y. Zheng, X. Zeng, W. Peng, Z. Wu, W. Su. Characterisation and classification of Citri Reticulatae Pericarpium varieties based on UHPLC-Q-TOF-MS/MS combined with multivariate statistical analyses. Phytochem. Anal. 30 (2019) 278-291. https://doi.org/10.1002/pca.2812.

[22] M. Della Greca, A. Molinaro, P. Monaco, L. Previtera. Two new lignan glucosides from *Arum italicum*. Heterocycles 9 (1993) 2081-2086. https://doi.org/10.3987/com-93-6424

[23] H. Zhao, M. Fan, X. Wu, H. Wang, J. Yang, N. Si, B. Bian. Chemical Profiling of the Chinese herb formula Xiao-Cheng-Qi decoction using liquid chromatography coupled with electrospray ionization mass spectrometry. J. Chromatogr. Sci. 51 (2013) 273-285. https://doi.org/10.1093/chromsci/bms138.

[24] R. Ben Said, A.I. Hamed, U.A. Mahalel, A.S. Al-Ayed, M. Kowalczyk, J. Moldoch, W. Oleszek, A. Stochmal. Tentative characterization of polyphenolic compounds in the male flowers of *Phoenix dactylifera* by liquid chromatography coupled with mass spectrometry and DFT. Int. J. Mol. Sci.; 18 (2017) 512. https://doi.org/10.3390/ijms18030512.

[25] A.M. El Sayed, S.M. Basam, E.A. El-Naggar, H.S. Marzouk, S. El-Hawary. LC–MS/MS and GC–MS profiling as well as the antimicrobial effect of leaves of selected *Yucca* species introduced to Egypt. Sci. Rep. 10 (2020) 17778. https://doi.org/10.1038/s41598-020-74440-y.

[26] X. Ma, Y. Wu, Y. Li, Y. Huang, Y. Liu, P. Luo, Z. Zhang. Rapid discrimination of *Notopterygium incisum* and *Notopterygium franchetii* based on characteristic compound profiles detected by UHPLC-QTOF-MS/MS coupled with multivariate analysis. Phytochem. Anal. 31 (2020) 355-365. https://doi.org/10.1002/pca.2902.

[27] M. Friščić, F. Bucar, K.H. Pilepić. LC-PDA-ESI-MS^n^ analysis of phenolic and iridoid compounds from *Globularia* spp. J. Mass Spectrom. 51 (2016) 1211-1236. https://doi.org/10.1002/jms.3844.

[28] M.A. Ghareeb, A.M. Saad, W.S. Ahmed, L.A. Refahy, S.M. Nasr. HPLC-DAD-ESI-MS/MS characterization of bioactive secondary metabolites from *Strelitzia nicolai* leaf extracts and their antioxidant and anticancer activities *in vitro*. Pharmacogn. Res. 10 (2018c) 368-378. https://doi.org/10.4103/pr.pr_89_18.

[29] W.H.B. Hassan, S. Abdelaziz, H.M. Al Yousef. Chemical composition and biological activities of the aqueous fraction of *Parkinsonea aculeata* L. growing in Saudi Arabia. Arab. J. Chem*.* [12 (2019](https://www.sciencedirect.com/science/journal/18785352/12/3)) 377-387. https://doi.org/10.1016/j.arabjc.2018.08.003.

[30] X. Zeng, W. Su, Y. Zheng, H. Liu, P. Li, W. Zhang, Y. Liang, Y. Bai, W. Peng, H. Yao. UFLC-Q-TOF-MS/MS-Based Screening and identification of flavonoids and derived metabolites in human urine after oral administration of *Exocarpium citri* grandis extract. Molecules. 23 (2018) 895. https://doi.org/10.3390/molecules23040895.

[31] A. Brito, J.E. Ramirez, C. Areche, B. Sepúlveda, M.J. Simirgiotis. HPLC-UV-MS profiles of phenolic compounds and antioxidant activity of fruits from three Citrus species consumed in Northern Chile. Molecules*.* 19 (2014) 17400-17421. https://doi.org/10.3390/molecules191117400.

[32] G. Chen, X. Li, F. Saleri, M. Guo. Analysis of flavonoids in *Rhamnus davurica* and its antiproliferative activities. Molecules. 21 (2016) 1275. https://doi.org/10.3390/molecules21101275.

[33] V. Milutinović, M. Niketić, L. Ušjak, D. Nikolić, A. Krunić, C. Zidornd, S. Petrović. Methanol extracts of 28 *Hieracium* species from the Balkan Peninsula-comparative LC-MS analysis, chemosystematic evaluation of their flavonoid and phenolic acid profiles and antioxidant potentials. Phytochem. Anal. 29 (2018) 30-47. <https://doi.org/10.1002/pca.2712>.

[34] I.M. Abu-Reidah, M.S. Ali-Shtayeh, R.M. Jamous, D. Arráez-Román, A. Segura-Carretero. HPLC-DAD-ESI-MS/MS screening of bioactive components from *Rhus coriaria* L. (Sumac) fruits. Food Chem. 166 (2015) 179-191. https://doi.org/10.1016/j.foodchem.2014.06.011.

[35] M. Sanz, B.F. de Simón, E. Cadahía, E. Esteruelas, A.M. Muñoz, T. Hernández, I. Estrellac, E. Pinto. LC-DAD/ESI-MS/MS study of phenolic compounds in ash (*Fraxinus excelsior* L. and *F. americana* L.) heartwood. Effect of toasting intensity at cooperage. J. Mass. Spectrom. 47 (2012) 905-918. https://doi.org/10.1002/jms.3040.

[36] D.H. Nguyen, B.T. Zhao, D.D. Le, K.Y. Kim, Y.H. Kim, Y.H. Yoon, J.Y. Ko, K.S. Woo, M.H. Woo. Phenolic constituents and their anti-inflammatory activity from *Echinochloa utilis* Grains. Nat. Prod. Sci. 22 (2016) 140-145.   https://doi.org/10.20307/nps.2016.22.2.140.

[37] M.A. Ghareeb, W.S. Ahmed, L.A. Refahy, A.M. Abdou, M.M. Hamed, M.S. Abdel-Aziz. Isolation and characterization of the bioactive phenolic compounds from *Morus alba* L. growing in Egypt*.* Pharmacologyonline. 3 (2016) 157-167.

[38] M.A. Ghareeb, H.A. Shoeb, H.M.F. Madkour, L.A. Refahy, M.A. Mohamed, A.M. Saad. Radical scavenging potential and cytotoxic activity of phenolic compounds from *Tectona grandis* (Linn.). Glob. J. Pharmacol. 7 (2013) 486-497. https://doi.org/10.5829/idosi.gjp.2013.7.4.8263.

[39] M.A. Ghareeb, H.A. Shoeb, H.M.F. Madkour, L.A. Refahy, M.A. Mohamed, A.M. Saad. Antioxidant and cytotoxic activities of flavonoidal compounds from *Gmelina arborea* (Roxb.). Glob. J. Pharmacol. 8 (2014) 87-97. https://doi.org/10.5829/idosi.gjp.2014.8.1.82194.
